# Antimicrobial Resistance, Serotypes, Virulence Gene Profiles, and Molecular Characterization of *Streptococcus suis* Isolated from Healthy Pigs in Thailand

**DOI:** 10.3390/antibiotics15070660

**Published:** 2026-07-03

**Authors:** Phirabhat Saengsawang, Pakpoom Tadee, Prapas Patchanee, Watcharapong Mitsuwan, Sumalee Boonmar, Hidenori Kabeya, Nattinee Kittiwan, Ravisa Warin, Ratchadaporn Boripun

**Affiliations:** 1 Department of Microbiology and Immunology, Faculty of Veterinary Medicine, Kasetsart University, 50 Ngamwongwan Road, Bangkok 10900, Thailand; fvetphsa@ku.ac.th; 2 Department of Food Animal Clinics, Faculty of Veterinary Medicine, Chiang Mai University, Chiang Mai 50100, Thailand or pakpoom.tad@cmu.ac.th (P.T.); prapas.pat@cmu.ac.th (P.P.); ravisa.wa@wu.ac.th (R.W.); 3 Akkhraratchakumari Veterinary College, Walailak University, Nakhon Si Thammarat 80160, Thailand; watcharapong.mi@wu.ac.th (W.M.); or sumalee.bo@wu.ac.th (S.B.); 4 One Health Research Center, Walailak University, Nakhon Si Thammarat 80160, Thailand; 5 Laboratory of Veterinary Food Hygiene, Department of Veterinary Medicine, College of Bioresource Sciences, Nihon University, 1866 Kameino, Fujisawa 252-0880, Kanagawa, Japan; kabeya.hidenori@nihon-u.ac.jp; 6 Veterinary Research and Development Center (Upper Northern Region), Hang Chat, Lampang 52190, Thailand; nattinee.k@dld.go.th

**Keywords:** antimicrobial susceptibility, serotyping, *Streptococcus suis*, whole genome sequencing, virulence genes

## Abstract

*Streptococcus suis* is an important cause of disease in pigs and an emerging source of severe human infection, with increasing concerns regarding antimicrobial resistance. This study assessed the occurrence and seroprevalence of *S. suis* in healthy pigs and characterized the antimicrobial susceptibility, serotypes, virulence associated genes, and genomic features of colonizing isolates. A total of 58 pigs (13 farm pigs and 45 abattoir pigs) were sampled. Among 176 presumptive isolates recovered by culture, 60 were confirmed as *S. suis* by polymerase chain reaction (PCR) and were subsequently analyzed. Antimicrobial susceptibility was determined by the disc diffusion method, selected resistance and virulence genes were detected by conventional PCR, and two representative isolates were subjected to whole-genome sequencing (WGS) and comparative genomic analyses. Seroprevalence was determined using an indirect ELISA detecting IgG antibodies against *S. suis* serotype 2 antigen. *S. suis* was confirmed in 60/176 presumptive isolates (34.1%), most frequently from nasal swabs and tonsils, whereas no blood isolates were detected. The seropositivity rate was 65.5%. High resistance rates were observed for ceftriaxone, ampicillin, cefepime, clindamycin, and tetracycline, and multidrug resistance was common. Tetracycline- and macrolide-associated resistance genes were frequently detected. Serotypes 2/1⁄2 predominated, whereas major classical invasive virulence genes were not detected, consistent with predominantly colonizing strains. Whole genome sequencing of two representative isolates confirmed species identity and revealed substantial genomic diversity, including a markedly larger genome in a tonsillar isolate, suggesting acquisition of accessory genetic elements. These findings demonstrate that healthy pigs can harbor genetically diverse and antimicrobial resistant *S. suis*, highlighting the importance of continued surveillance to support antimicrobial stewardship and reduce zoonotic risk.

## 1. Introduction

*Streptococcus suis* (*S. suis*) is a Gram-positive coccus that commonly colonizes pigs and is recognized as an important pig pathogen responsible for meningitis, septicemia, arthritis, and sudden death, leading to substantial economic losses in the pig industry [1,2,3]. Its natural reservoir lies primarily in the upper respiratory tract, particularly the tonsils and nasal cavities as well as in the genital and alimentary tracts of pigs [4]. Transmission between herds mainly occurs through the movement of asymptomatic carrier pigs harboring the bacterium [5]. Beyond its veterinary significance, *S. suis* has also emerged as a zoonotic pathogen capable of causing severe human diseases, including meningitis, sepsis, septic shock, infective endocarditis, and septic arthritis [6]. The public health burden is particularly pronounced in Southeast Asia, notably in Thailand, where recurrent outbreaks and sporadic human infections have been linked to pig production practices and specific dietary habits [7,8]. In Thailand, the consumption of traditional raw pork dishes, particularly “larb moo dip” (raw minced pork salad), has been identified as a major risk factor for human *S. suis* infection. Such cultural dietary practices facilitate direct exposure to the pathogen and contribute significantly to the high incidence of zoonotic cases reported in the region. These dual impacts on animal and human health underscore the need for integrated surveillance encompassing antimicrobial susceptibility, virulence characteristics, and population structure.

Antimicrobial resistance (AMR) in *S. suis* poses growing challenges for both veterinary treatment and public health. National surveillance programs in Thailand have reported persistently high levels of resistance to macrolides and tetracyclines, while β-lactams and phenicols generally remain effective; however, resistance patterns vary across geographic regions, time periods, and bacterial lineages [9,10]. Continuous monitoring of AMR in pig populations is therefore critical to support antimicrobial stewardship and mitigate zoonotic risks along the farm to fork continuum. Despite decades of research, no broadly effective vaccine against *S. suis* is currently available. Existing bacterin based vaccines provide only partial, serotype specific protection [9,10]. Consequently, antimicrobial agents remain the primary tools for disease treatment and control. Investigations of *S. suis* isolates from diseased and healthy pigs across different countries have shown that β-lactams, tetracyclines, sulfonamides, and macrolides are the most frequently used antimicrobials for prevention and therapy. Nevertheless, considerable variation in antimicrobial resistance has been observed between regions, serotypes, and time periods. Widespread resistance to commonly used agents has been driven largely by the indiscriminate and excessive use of antibiotics in pig production [10,11,12].

*S. suis* strains are classified serologically according to their capsular polysaccharide (CPS) antigens [13]. To date, 35 serotypes have been identified [14], although several (including serotypes 20, 22, 26, and 32–34) have been reassigned to other *Streptococcus* species based on molecular taxonomic analyses [15,16]. Among these, serotype 2 is the most prevalent and virulent worldwide, followed by serotype 9, which is also frequently associated with disease in pigs, particularly in Europe [17]. Virulence among *S. suis* isolates varies widely between serotypes and even among strains within the same serotype. In serotypes 1 and 2, virulence is mainly associated with three key proteins: muramidase-released protein (MRP), extracellular factor (EF), and suilysin. Strains positive for MRP and EF are typically virulent, whereas those lacking these proteins are usually less virulent or avirulent and often isolated from the tonsils of healthy pigs [17]. However, these markers are not universally predictive of virulence. For instance, most serotype 2 isolates from Canada lack these proteins [18], while the majority of European isolates express them [19]. EF has been linked to virulence in serotypes 1/2 and 14 but not in serotypes 7 and 9, where instead a variant of MRP appears to contribute to pathogenicity [20].

The distribution of virulence-associated genes and the genetic diversity of circulating *S. suis* populations are key determinants of pathogenicity, transmission dynamics, and epidemiology. However, data on antimicrobial susceptibility patterns, virulence gene profiles, and genetic diversity of *S. suis* isolates from pig farms and slaughterhouses in Thailand remain limited. Comprehensive characterization of these features is essential to guide rational antimicrobial use, improve disease control strategies, and reduce zoonotic risk along the pork production chain. The objectives of this study were to determine prevalence and seroprevalence of *S. suis* isolated from pigs, and to characterize their antibiotic susceptibility, presence of virulent genes, and genomic features from draft whole genome.

## 2. Results

### 2.1. Prevalence of S. suis and Seroprevalence of S. suis Serotype 2

A total of 161 biological samples were collected from 58 pigs, comprising 58 nasal swabs, 58 blood samples, and 45 tonsil swabs. Following culture on Todd Hewitt agar, 176 presumptive isolates with morphological characteristics consistent with *S. suis* were recovered, including 89 isolates from nasal swabs, 76 from tonsil swabs, and 11 from blood samples. Because multiple presumptive colonies could be recovered from a single sample, the number of presumptive isolates exceeded the number of collected samples. These presumptive isolates were subsequently subjected to PCR confirmation.

Of the 176 presumptive isolates, 60 (34.1%; 95% CI: 27.4–41.3%) were confirmed as *S. suis* by PCR. Among the 76 presumptive isolates recovered from tonsil swabs, 26 (34.2%; 95% CI: 24.3–45.5%) were PCR positive for *S. suis*. Similarly, 34 of 89 presumptive isolates recovered from nasal swabs (38.2%; 95% CI: 28.8–48.5%) were confirmed as *S. suis*, whereas 55 isolates (61.8%) were PCR negative. None of the 11 presumptive isolates recovered from blood samples were confirmed as *S. suis* (0%; 95% CI: 0–25.9%). All PCR confirmed isolates (*n* = 60) were included in subsequent serotyping, antimicrobial susceptibility testing, virulence gene detection, and whole-genome sequencing analyses.

In addition, 58 serum samples were analyzed for immunoglobulin G (IgG) antibodies against *S. suis* serotype 2. The seropositivity rate was 65.5% (95% CI: 51.9–77.5%). The overall sample-to-positive (S/P) ratio was 0.35 ± 0.29, with mean S/P ratios of 0.48 ± 0.27 and 0.10 ± 0.04 in the seropositive and seronegative groups, respectively. The distribution of PCR-confirmed *S. suis* isolates recovered from each sample type is presented in Table 1.

### 2.2. Presence of Antimicrobial Susceptibility

**Figure 1:**
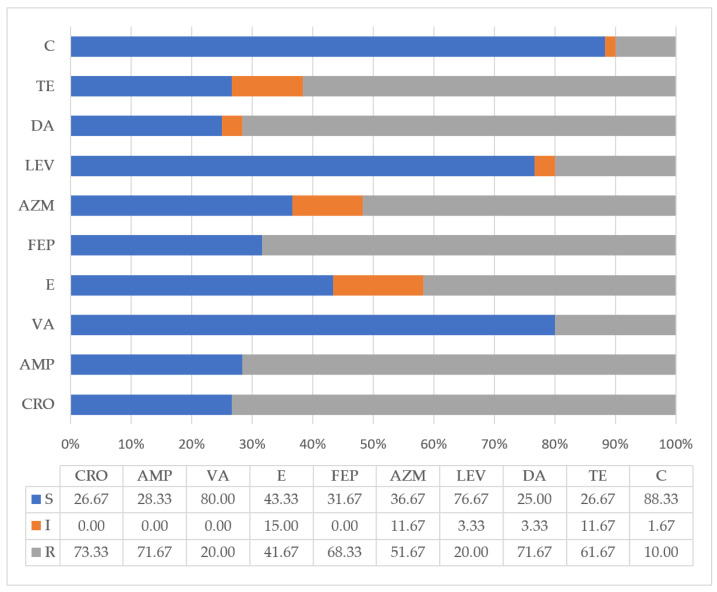
Antimicrobial susceptibility profiles of *S. suis* isolates (n = 60) determined by the Kirby–Bauer disc diffusion method, showing the percentages of susceptible (S), intermediate (I), and resistant (R) isolates for each antimicrobial agent.

Antimicrobial susceptibility testing (AST) was performed on all 60 PCR-confirmed *S. suis* isolates using the Kirby–Bauer disk diffusion method. The proportions of resistant (R) isolates for each antimicrobial agent are shown in Figure 1. The highest resistance rates were observed for ceftriaxone (CRO, 73.33%), ampicillin (AMP, 71.67%), clindamycin (DA, 71.67%), cefepime (FEP, 68.33%), and tetracycline (TE, 61.67%). In contrast, lower resistance rates were observed for chloramphenicol (C, 10.00%), vancomycin (VA, 20.00%), and levofloxacin (LEV, 20.00%). Resistance to erythromycin (E, 41.67%) and azithromycin (AZM, 51.57%) was moderate, while intermediate and susceptible categories are not presented here to improve clarity and focus on resistance patterns relevant to antimicrobial resistance surveillance (Figure 1).

Antimicrobial susceptibility testing was performed on isolates obtained from nasal swabs (n = 34) and tonsil swabs (n = 26). High resistance rates were observed against several antimicrobial agents in isolates from both sampling sites. Among nasal swab isolates, the highest resistance rates were detected against ceftriaxone (CRO; 76.47%), ampicillin (AMP; 73.53%), cefepime (FEP; 70.59%), and clindamycin (DA; 67.65%). Moderate resistance was observed for tetracycline (TE; 55.88%), azithromycin (AZM; 44.12%), and erythromycin (E; 35.29%), whereas lower resistance rates were found for vancomycin (VA; 23.53%), levofloxacin (LEV; 20.59%), and chloramphenicol (C; 8.82%). Intermediate susceptibility was observed for E (20.59%), AZM (20.59%), TE (14.71%), DA (5.88%), LEV (2.94%), and C (2.94%). Among tonsil swab isolates, the highest resistance rates were observed for ceftriaxone (CRO; 69.23%), ampicillin (AMP; 69.23%), tetracycline (TE; 69.23%), cefepime (FEP; 65.38%), azithromycin (AZM; 61.54%), erythromycin (E; 50.00%), and clindamycin (DA; 76.92%). Lower resistance rates were detected for levofloxacin (LEV; 19.23%), vancomycin (VA; 15.38%), and chloramphenicol (C; 11.54%). Intermediate susceptibility was uncommon and observed only for E (7.69%), LEV (3.85%), and TE (7.69%). Notably, chloramphenicol, vancomycin, and levofloxacin showed the highest susceptibility rates, ranging from 76.47% to 88.46% in isolates from both sampling sites (Table 2). Multidrug resistance was commonly observed among the isolates, with 39 isolates showing resistance to three or more antimicrobial classes. The detailed MDR patterns are presented in Table S1.

### 2.3. Distribution of Antimicrobial Resistance Gene

A total of 30 isolates were screened for the presence of antimicrobial resistance genes, including *tetO*, *tetM*, *ermA*, *ermB*, and *mefA*. The most frequently detected gene was *tetM*, identified in 27/30 isolates (90.00%), followed by *tetO* in 24/30 isolates (80.00%). The macrolide resistance genes *mefA* and *ermB* were detected in 23/30 (76.67%) and 21/30 (70.00%) isolates, respectively, while *ermA* was present in 17/30 isolates (56.67%). Negative detection rates were 10.00% (3/30) for *tetM*, 20.00% (6/30) for *tetO*, 26.67% (8/30) for *mefA*, 30.00% (9/30) for *ermB*, and 46.67% (14/30) for *ermA*. Overall, tetracycline resistance genes (*tetM* and *tetO*) were more prevalent than macrolide resistance genes among the tested isolates (Table 3). Differences were analyzed using the Fisher Freeman Halton exact test in R version 4.3.0. A *p*-value < 0.05 was considered statistically significant.

### 2.4. Detection of Molecular Typing

**Figure 2:**
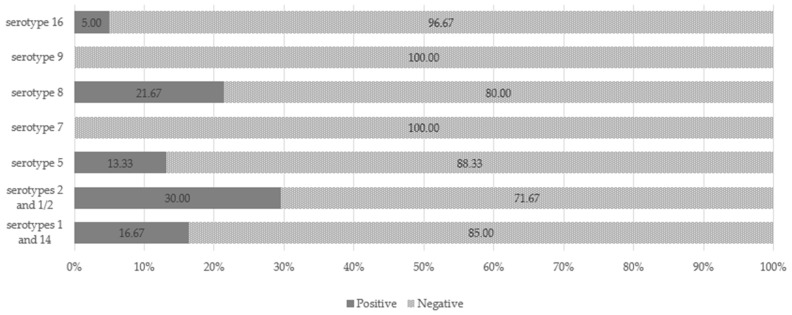
Proportion of positive and negative isolates by serotype (*n* = 60).

The serotype distribution of the 60 *S. suis* isolates revealed that serotype 2/1/2 was the predominant serotype (30.0%), followed by serotype 8 (21.7%), serotypes 1 and 14 (16.7%), serotype 5 (13.3%), and serotype 16 (5.0%). These findings indicate that serotype 2/1/2 was the most frequently identified serotype among healthy pigs in the present study (Figure 2).

The spreading of *S. suis* serotypes varied according to the site of isolation. Among nasal swab isolates *(n* = 34), serotype 2 and 1/2 was the most prevalent (35.29%), followed by serotype 8 (26.47%), serotype 5 (17.64%), serotypes 1 and 14 (11.76%), and serotype 16 (2.94%). In tonsil swab isolates (*n* = 24), serotypes 1 and 14 and serotype 2 and 1/2 were equally predominant (23.07% each), followed by serotype 8 (15.38%), serotype 5 (7.69%), and serotype 16 (7.69%). No isolates belonging to serotypes 7 or 9 were detected in either sampling site. Blood samples (*n* = 11) yielded no *S. suis* isolates. Overall, serotype 2 and 1/2 was the most frequently identified serotype (30%), followed by serotype 8 (21.67%), serotypes 1 and 14 (16.67%), serotype 5 (13.33%), and serotype 16 (1.67%). These findings indicate that serotype 2 and 1/2 predominated in both nasal and tonsillar samples within the studied population. Table 4 presents the distribution of molecular serotyping of *S. suis* isolated from collected samples.

### 2.5. Detection of Virulence Genes

Among the 60 *S. suis* isolates examined (34 nasal and 26 tonsillar isolates), *bay046* was the most prevalent virulence associated gene, detected in 56 isolates (93.3%), with similarly high frequencies in nasal (94.1%) and tonsillar (92.3%) isolates. The *arcA* gene was identified in 42 isolates (70.0%) and was more frequently detected in nasal isolates (79.4%) than in tonsillar isolates (57.7%). In contrast, the *mrp* gene was detected only sporadically, occurring in three isolates (5.0%).

Overall, the virulence gene profiles were dominated by genes associated with colonization and metabolism, particularly *bay046* and *arcA*, whereas genes commonly associated with highly virulent strains were rarely detected. The prevalence of virulence associated genes according to sample type is presented in Table 5.

The distribution of virulence-associated genes among *S. suis* serotypes (*n* = 60) demonstrated marked variation. The *bay046* gene was predominantly detected in serotypes 2 and 1/2 (30.00%), followed by serotype 8 (21.67%), serotypes 1 and 14 (15.00%), serotype 5 (13.33%), and serotype 16 (5.00%), whereas it was not identified in serotypes 7 and 9 (0%). Similarly, the *arcA* gene was most frequently detected in serotypes 2 and 1/2 (21.67%), followed by serotype 8 (11.67%), serotypes 1 and 14 (10.00%), serotype 5 (6.67%), and serotype 16 (1.67%); no *arcA*-positive isolates were observed in serotypes 7 and 9. The *mrp* gene was detected at a low frequency (1.67%) and was restricted to serotypes 2 and 1/2. In contrast, *epf*, *hly*, and *sly* were not detected in any serotype (0%). Overall, serotypes 2 and 1/2 exhibited the highest proportion of virulence associated genes, particularly *bay046* and *arcA*, while several serotypes lacked detectable virulence determinants (Table 6).

### 2.6. Whole Genome Sequence

Whole genome sequencing (WGS) was performed for two representative *S. suis* isolates obtained from a nasal swab (NS05) and a tonsil swab (T14). The draft genome assembly of NS05 comprised 92 contigs with a total genome length of 2,183,810 bp and a GC content of 41.03%. In contrast, isolate T14 consisted of 78 contigs with a larger total genome size of 3,846,008 bp and a GC content of 39.67%. No plasmids were detected in either isolate. Assembly quality metrics indicated that T14 showed greater assembly contiguity, with a lower contig L50 (7 vs. 12) and a higher contig N50 value (168,725 bp vs. 68,551 bp) compared to NS05. Overall, the tonsil-derived isolate demonstrated a more contiguous genome assembly and a larger genome size than the nasal-derived isolate (Table 7).

WGS analysis revealed that both nasal and tonsillar isolates carried diverse AMR determinants representing multiple resistance mechanisms, including antibiotic inactivation, target modification, target protection, target replacement, and regulatory modulation. The nasal isolate NS05 harbored several aminoglycoside-modifying enzyme genes, including *APH(3′)-III*, *APH*(*3′)-IV*, *APH(3′)-VI*, *APH(3′)-VII*, and members of the *CatA8* family, whereas the tonsillar isolate T14 carried *ANT(6)-I* and *ANT(9)-I*, also associated with aminoglycoside resistance. Both isolates possessed conserved intrinsic resistance-associated genes encoding antibiotic targets in susceptible species, including *alr*, *ddl*, *EF-G*, *EF-Tu*, *folA*, *dfr*, *folP*, *gyrA*, *gyrB*, *iso-tRNA*, *kasA*, *murA*, *rpoB*, *rpoC*, *S10p*, and *S12p*. Additionally, both isolates carried antibiotic target modifying enzymes *ermB* and *rlmA(II)*, which are associated with macrolide lincosamide resistance. Tetracycline resistance determinants differed between isolates: NS05 harbored *tetW*, whereas T14 carried *tetO*, both encoding ribosomal protection proteins. The antibiotic target replacement gene *fabK* was detected in both isolates. The gene *gidB*, associated with resistance via gene absence or modification, was also present in both genomes. Regarding mechanisms affecting cell envelope properties, NS05 carried *pgsA*, while T14 harbored *gdpD*, *mprF*, and *pgsA*, genes implicated in altering cell wall charge and contributing to reduced susceptibility to certain antimicrobial agents. Furthermore, both isolates contained the regulatory genes *liaF*, *liaR*, and *liaS*, which modulate the expression of cell envelope stress and antibiotic resistance responses (Table 8).

### 2.7. Analysis Based on the Concatenated Nucleotide Sequences of the Seven Housekeeping Genes

**Figure 3:**
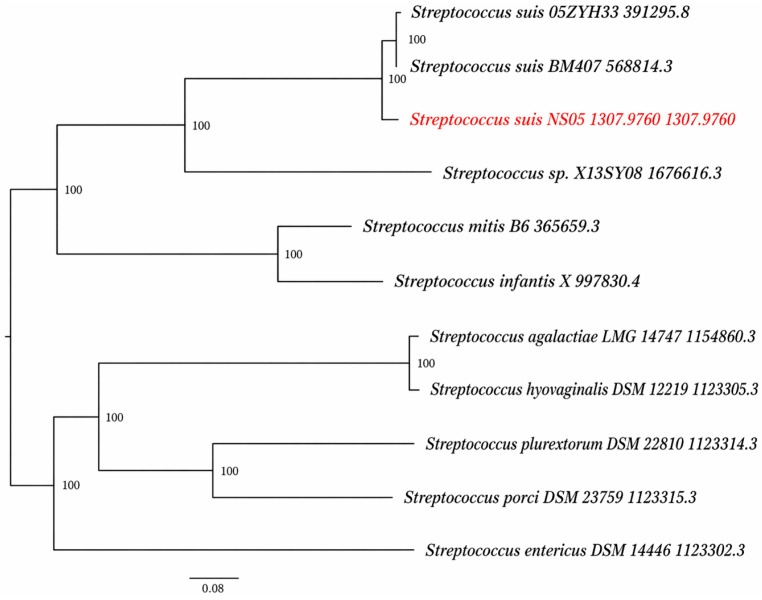
Phylogenetic tree based on the concatenated nucleotide sequences of the seven housekeeping genes of *S. suis* strains obtained in this study (Nasal Swab Sample; NS05).

**Figure 4:**
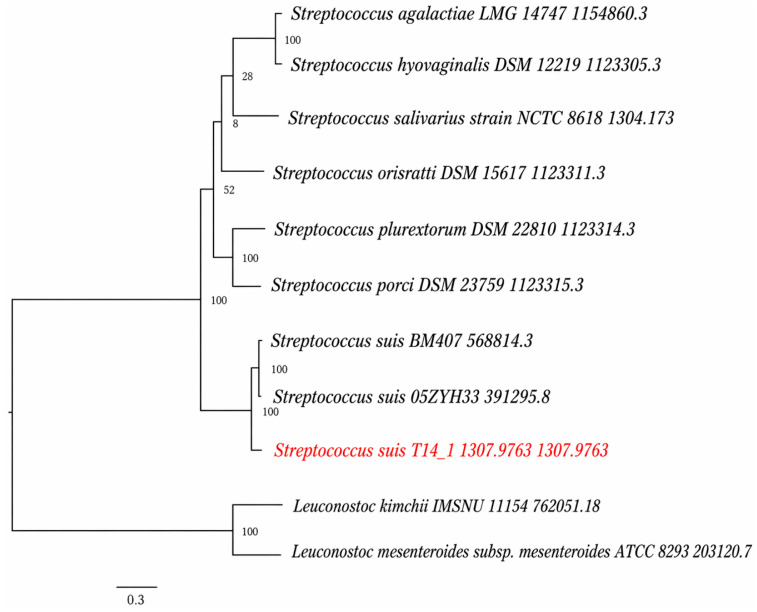
Phylogenetic tree based on the concatenated nucleotide sequences of the seven housekeeping genes of *S. suis* strains obtained in this study (Tonsil Swab Sample; T14).

Phylogenetic analysis confirmed that both the nasal swab isolate (NS05) and the tonsillar swab isolate (T14) clustered within the *S. suis* lineage. In both trees, the study isolates grouped tightly with the reference *S. suis* strains BM407 and 05ZYH33, forming a well-supported monophyletic clade with 100% bootstrap support. The short branch lengths separating NS05 and T14 from these reference strains indicate a high degree of genetic similarity. In the first tree, NS05 clustered directly with *S. suis* BM407 and 05ZYH33, clearly separated from other closely related Streptococcus species, including *Streptococcus mitis*, *Streptococcus infantis*, *Streptococcus agalactiae*, *Streptococcus hyovaginalis*, *Streptococcus plurextorum*, *Streptococcus porci*, and *Streptococcus entericus*. All major internal nodes were supported by bootstrap values of 100%, indicating strong robustness of the inferred topology. Similarly, in the second tree, the tonsillar isolate T14 grouped within the same *S. suis* clade as BM407 and 05ZYH33, again with 100% bootstrap support. This cluster was clearly distinct from other streptococcal species, including *Streptococcus agalactiae*, *Streptococcus hyovaginalis*, *Streptococcus salivarius*, *Streptococcus orisratti*, *Streptococcus plurextorum*, and *Streptococcus porci*. *Leuconostoc kimchii* and *Leuconostoc mesenteroides* were included as outgroup taxa to root the tree, and were clearly separated from the Streptococcus clade. Phylogenetic analysis of *S. suis* isolate NS05 and isolate T14 presents in Figure 3 and Figure 4, respectively.

### 2.8. Genome Mapping

Whole genome sequencing of the nasal (NS) isolate *S. suis* NS05 yielded a single circular chromosome of approximately 2.1 Mbp with a GC content consistent with previously reported *S. suis* genomes (~41–42%). GC skew analysis demonstrated a typical bidirectional replication pattern with clearly defined origin and terminus regions. Genome annotation identified coding sequences distributed on both strands, including core housekeeping genes involved in replication, transcription, translation, cell division, and stress response, as well as multiple rRNA operons and tRNA genes. A proportion of predicted coding sequences were annotated as hypothetical proteins. Resistome analysis using the CARD database identified several antimicrobial resistance determinants, including *ermB* (macrolide resistance), *APH(3′)-IIIa* (aminoglycoside resistance), catA8 (chloramphenicol resistance), *tet(O/W/32/O)* (tetracycline resistance), and *vanY* within a *vanB*-associated cluster. Additionally, the presence of *patA* and *patB* suggests efflux-mediated resistance mechanisms. The clustering of several resistance genes within the chromosome indicates potential acquisition through mobile genetic elements. Overall, the nasal isolate NS05 harbors multiple antimicrobial resistance determinants, highlighting the upper respiratory tract as a potential reservoir of multidrug resistant *S. suis* strains.

*S. suis* T14 generated a single circular chromosome of approximately 3.5 Mbp with a GC content consistent with the species (~41–42%). GC content and GC skew analyses demonstrated a typical bidirectional replication pattern with clearly defined origin and terminus regions. Genome annotation identified coding sequences distributed across both strands, including core housekeeping genes involved in DNA replication, transcription, translation, metabolism, and stress response. Multiple tRNA and rRNA genes were detected, and a CRISPR locus was identified, suggesting potential adaptive immunity against mobile genetic elements. A number of predicted coding sequences were annotated as hypothetical proteins.

**Figure 5:**
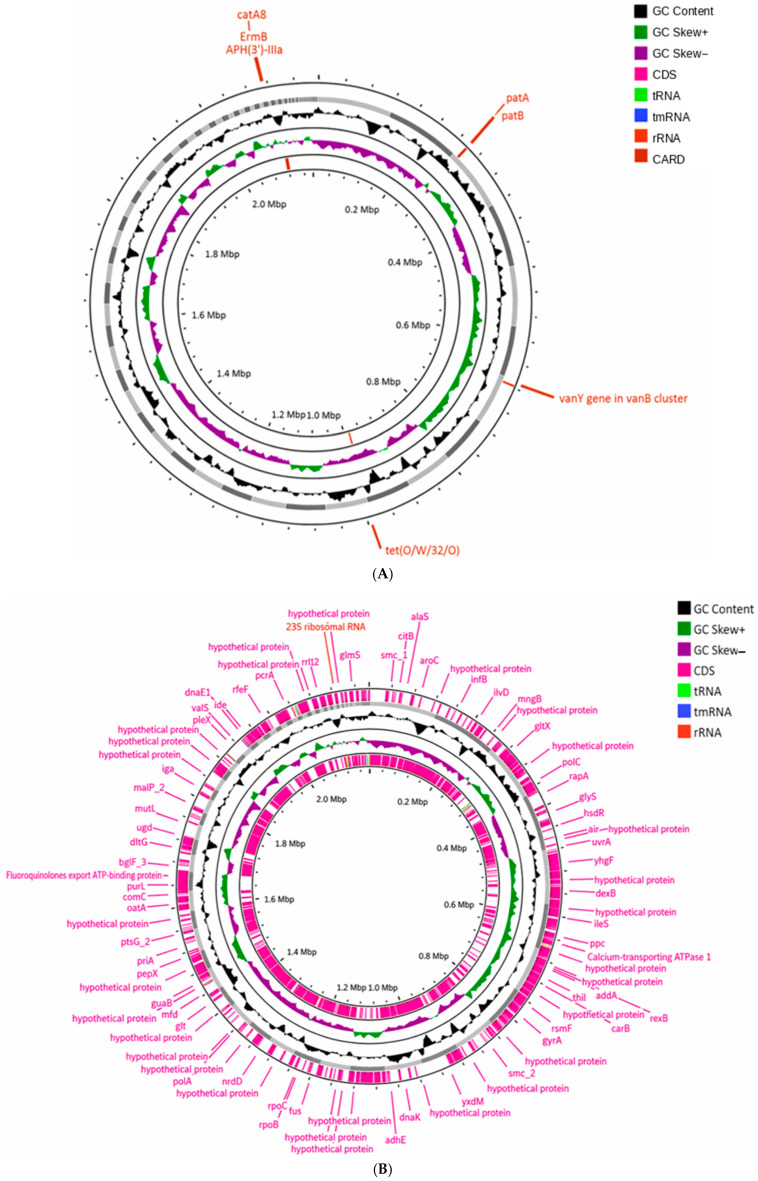
Circular genome maps of *S. suis* NS05. (**A**) Circular representation of the complete genome showing GC content, GC skew, coding sequences (CDSs), and RNA genes (tRNA, tmRNA, and rRNA). (**B**) Circular representation of the complete genome highlighting the distribution of antimicrobial resistance determinants identified using the Comprehensive Antibiotic Resistance Database (CARD), together with GC content, GC skew, coding sequences, and RNA genes.

**Figure 6:**
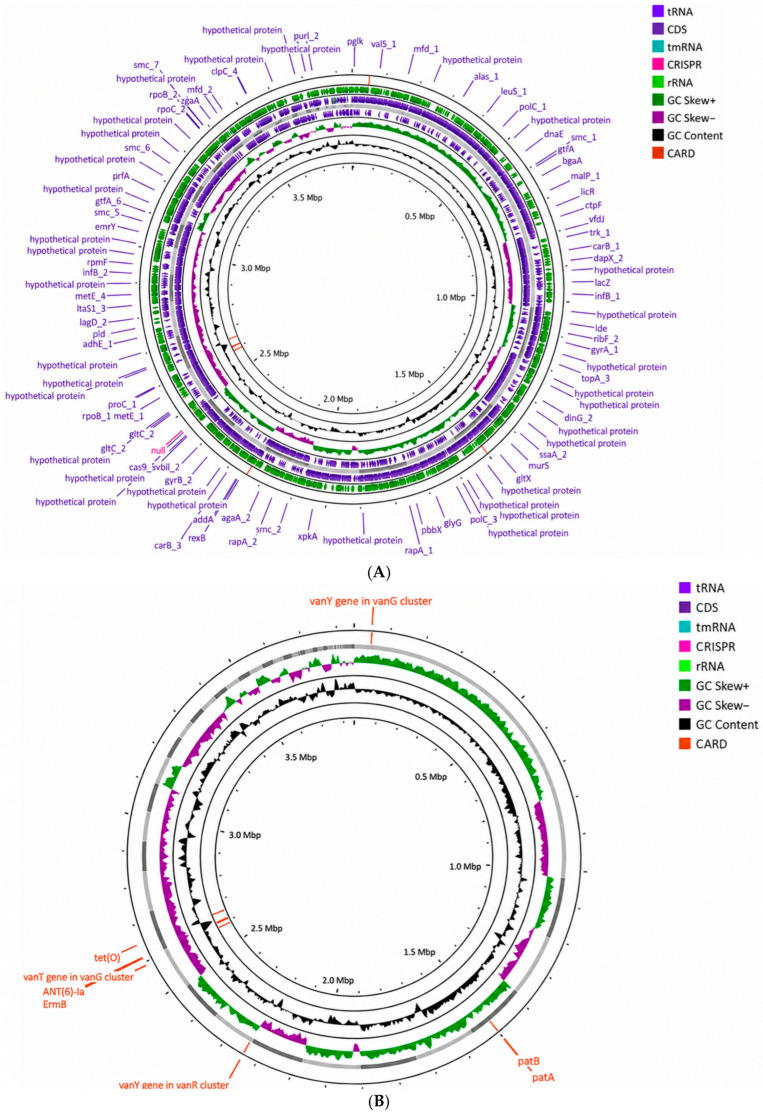
Circular genome maps of *S. suis* T14. (**A**) Circular representation of the complete genome showing GC content, GC skew, coding sequences (CDSs), and RNA genes (tRNA, tmRNA, and rRNA). (**B**) Circular representation of the complete genome highlighting the distribution of antimicrobial resistance determinants identified using the Comprehensive Antibiotic Resistance Database (CARD), together with GC content, GC skew, coding sequences, and RNA genes.

Resistome analysis using the CARD database revealed multiple antimicrobial resistance determinants. These included *ermB* (macrolide resistance), *tetO* (tetracycline resistance), and *ANT(6)-Ia* (aminoglycoside resistance). Notably, vancomycin-associated genes were detected, including *vanY* within a *vanG* cluster and an additional *vanY* gene associated with a *vanB* cluster, as well as *vanT* in the *vanG* cluster. The presence of these glycopeptide resistance-associated genes suggest acquisition of van gene clusters, although the completeness of the operons and their phenotypic expression would require further confirmation. Efflux-associated genes *patA* and *patB* were also identified, indicating potential contribution to multidrug resistance (Figure 5 and Figure 6).

### 2.9. Comparative Analysis of Capsular Polysaccharide Biosynthesis (cps) Gene Clusters

**Figure 7:**
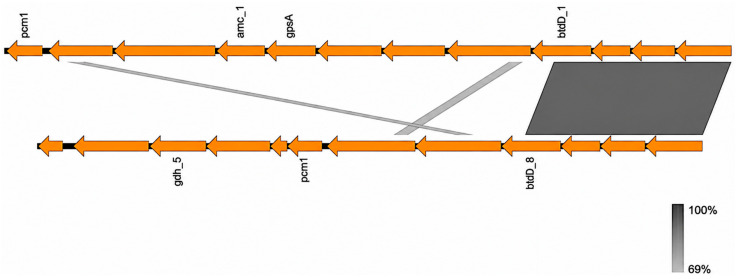
Comparison of capsular polysaccharide biosynthesis gene clusters between *S. suis* NS05 (**top**) and T14 (**bottom**).

Whole genome sequencing based comparative analysis of the capsular polysaccharide biosynthesis (cps) locus between *S. suis* NS05 and *S. suis* T14 revealed a highly conserved overall gene organization with localized variations. CDS-level synteny analysis demonstrated that most genes within the cps cluster were collinear between the two strains, indicating structural conservation of the capsule biosynthesis region. The upstream regulatory and conserved genes showed strong sequence similarity (69–100% nucleotide identity), suggesting preservation of core capsule biosynthesis machinery. However, notable differences were observed within the central variable region of the cps locus, where gene content and sequence divergence were evident. Specifically, variations were identified in glycosyltransferase-associated genes (e.g., *gtrA* homologs) and adjacent biosynthesis-related genes, which are typically responsible for serotype-specific polysaccharide structure. Additionally, a distinct genomic segment was present in one strain but absent or divergent in the other, indicating potential recombination or horizontal gene transfer events within the cps region. Such structural variation in the capsule locus likely contributes to differences in capsular composition and may influence antigenicity, virulence potential, and host immune evasion (Figure 7).

### 2.10. Functional Annotation of Genes Within the Capsular Polysaccharide Biosynthesis Cluster

**Figure 8:**
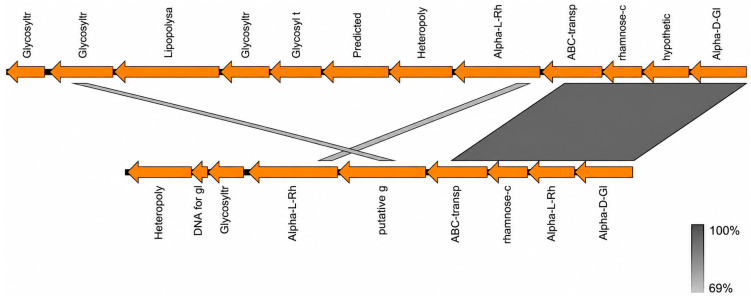
Comparison of functional annotation of genes within the capsular polysaccharide gene cluster between *S. suis* NS05 (**top**) and T14 (**bottom**).

Functional annotation of the capsular polysaccharide (*cps*) gene cluster identified multiple genes encoding enzymes and transport-related proteins involved in capsule biosynthesis and export. The predicted gene products included several glycosyltransferases, lipopolysaccharide-associated proteins, heteropolysaccharide biosynthesis proteins, and sugar-modifying enzymes, indicating a complete pathway for polysaccharide assembly. Notably, genes encoding rhamnose-related enzymes (e.g., alpha-l-rhamnosyltransferase), mannose-associated proteins, and other glycosyltransferases were detected, suggesting that the capsule structure likely incorporates multiple sugar residues. The presence of ABC transporter components within the cluster supports a Wzx/Wzy or ABC transporter-dependent capsule export mechanism. Additional genes annotated as hypothetical or predicted proteins were identified within the cluster, which may represent strain-specific or poorly characterized components contributing to capsular structure or regulation. Synteny analysis demonstrated conserved gene orientation across strains, although variations in gene content and sequence similarity (69–100%) were observed, particularly in the central biosynthetic region, consistent with capsular diversity (Figure 8).

### 2.11. Average Nucleotide Identity (ANI)

**Figure 9:**
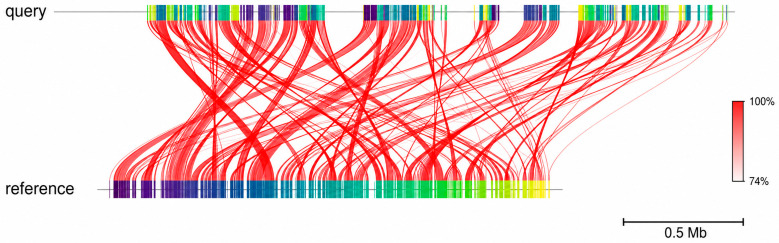
Whole-Genome Average Nucleotide Identity (FastANI) Alignment Between NS05 (Reference) and T14 (Query).

Whole-genome relatedness between isolate T14 and the reference genome NS05 was assessed using FastANI. The analysis yielded an average nucleotide identity (ANI) of 95.06%, based on 530 orthologous fragment matches out of 1250 query fragments. The ANI value lies at the accepted species delineation threshold (95–96%), indicating that T14 belongs to the same species as NS05 but represents a distinct genomic lineage. The proportion of orthologous matches suggests conservation of the core genome alongside the presence of accessory genomic regions (Figure 9).

## 3. Discussion

In the present study, *S. suis* was detected in 34.1% of samples, with the highest positivity observed in nasal swabs followed by tonsillar swabs, while no isolates were recovered from blood samples. These findings are consistent with the known ecology of *S. suis,* which primarily colonizes the upper respiratory tract particularly the tonsils and nasal cavities of pigs [9]. A similar nasal carriage rate (34.2%) was reported in clinically healthy pigs in Jiangxi Province, China [21], suggesting that the prevalence observed in this study falls within the expected range for asymptomatic populations. Tonsillar carriage has also been identified as a major reservoir for herd transmission [22]. The absence of systemic isolates aligns with the opportunistic nature of *S. suis*, where invasive disease typically occurs under predisposing factors such as stress or co-infections [23].

High resistance rates were observed for ceftriaxone, ampicillin, clindamycin, cefepime, and tetracycline, while chloramphenicol, vancomycin, and levofloxacin showed comparatively higher susceptibility. High resistance to tetracycline and macrolide lincosamide agents has been widely reported globally [24]. Similar resistance patterns have been documented among isolates from healthy pigs in China [21] and Thailand [25]. In the United States, tetracycline resistance was also the most prevalent phenotype [26]. These findings likely reflect extensive use of these antimicrobial classes in pig production systems. Although β-lactams have historically remained effective against *S*. *suis*, emerging resistance has been increasingly reported. Increased penicillin MIC values associated with alterations in penicillin-binding proteins have been described in Italy [27], and reduced susceptibility to veterinary β-lactams has also been noted in Thailand [25]. The emergence of β-lactam resistance is therefore of growing concern. It is also important to note that some antimicrobial susceptibility patterns may be influenced by intrinsic characteristics of *S. suis*. In particular, reduced susceptibility to certain antimicrobial classes, such as aminoglycosides, has been reported and is associated with limited drug uptake rather than acquired resistance mechanisms. Therefore, phenotypic resistance observed in this study should be interpreted with consideration of both intrinsic and acquired resistance mechanisms. Multidrug resistance (MDR) observed in this study is consistent with reports from Thailand [24] and France, where antimicrobial resistance genes were frequently associated with mobile genetic elements [28]. Horizontal gene transfer may play a significant role in the dissemination of resistance within pig populations. The detection of *tetM* and *tetO*, as well as *ermB* among the subset of isolates examined, is in agreement with global reports of tetracycline and macrolide resistance determinants. However, as antimicrobial resistance genes were assessed in a subset of isolates, these findings should be interpreted cautiously, as selection bias may limit the ability to infer true population-level prevalence. Macrolide resistance genes are commonly associated with mobile genetic elements, facilitating horizontal transfer [28]. Overall, these results suggest that antimicrobial usage practices likely contribute to selective pressure shaping the *S. suis* resistome in pig populations.

Serotypes 2 and 1/2 were the most frequently detected serotypes in this study. However, the PCR assay employed could not differentiate between serotype 2 and serotype 1/2; therefore, these serotypes were reported together. Serotype 2 is recognized worldwide as one of the most important serotypes associated with disease in pigs and human infections [23], and has been implicated in major outbreaks in Asia [29]. In Thailand, serotype 2 has also been reported as a predominant serotype among diseased pigs [25]. Similarly, serotype 8 has been identified among common serotypes in swine populations in other countries, including the United States [26]. Most isolates carried the colonization associated genes *bay046* and *arcA*, whereas classical virulence associated genes, including *epf*, *sly*, and *hly*, were not detected. Previous studies have reported that *epf*, *mrp*, and *sly* are commonly associated with invasive *S. suis* strains [30], and invasive serotype 2 isolates from Thailand frequently harbor multiple virulence-associated genes [31]. The predominance of *bay046* and *arcA*, together with the absence of major virulence markers, suggests that the isolates recovered in the present study primarily represent colonizing populations rather than highly invasive strains. Nevertheless, isolates belonging to the serotypes 2 and 1/2 group exhibited the highest frequency of virulence associated genes among the serotypes examined, indicating that their pathogenic potential should not be overlooked. A recent systematic review highlighted that the zoonotic potential of *S. suis* is influenced not only by serotype but also by genetic lineage and the combination of virulence determinants present within individual strains [32]. Therefore, the detection of serotypes 2 and 1/2 alone should not be interpreted as evidence of high virulence or zoonotic potential. However, the coexistence of zoonotically important serotypes and multidrug-resistant isolates in healthy pigs may contribute to the maintenance and dissemination of antimicrobial resistance and virulence-associated traits within pig populations. Continued surveillance integrating antimicrobial susceptibility testing, serotyping, virulence characterization, and genomic analyses is therefore warranted to improve understanding of the epidemiology and public health significance of *S. suis* in Thailand.

Whole genome sequencing confirmed that both NS05 (nasal isolate) and T14 (tonsillar isolate) belong to *S. suis*, as demonstrated by robust phylogenetic clustering with reference strains such as BM407 and 05ZYH33 and strong bootstrap support (100%). The short branch lengths observed indicate high genomic similarity within the *S. suis* lineage, supporting accurate species level identification. Phylogenetic separation from other streptococcal species further excludes misclassification and is consistent with previous genomic studies demonstrating clear monophyletic clustering of *S. suis* relative to closely related taxa [33,34]. Genome expansion in *S. suis* has been linked to horizontal gene transfer, integration of mobile genetic elements, prophages, and integrative conjugative elements (ICEs) [35,36,37,38]. The tonsillar niche, characterized by dense microbial communities and frequent genetic exchange, may facilitate such acquisition events. The higher assembly contiguity of T14 (lower L50, higher N50) supports that the larger genome size likely reflects biological reality rather than assembly artifact. Both isolates harbored multiple antimicrobial resistance (AMR) determinants representing diverse resistance mechanisms, including antibiotic inactivation, target modification, target protection, and regulatory modulation. Aminoglycoside resistance genes differed between isolates, with NS05 carrying multiple APH variants and T14 harboring ANT genes. Aminoglycoside modifying enzymes are widely distributed in *S. suis* and contribute significantly to resistance phenotypes observed in pig production systems [25,39]. The presence of different aminoglycoside resistance genes in isolates from distinct anatomical sites suggests independent acquisition events and highlights ongoing genetic exchange within porcine populations.

Macrolide lincosamide resistance mediated by *ermB* was detected in both isolates. The *ermB* gene is among the most prevalent resistance determinants in *S. suis* globally and is frequently associated with mobile genetic elements [25,26]. Its widespread distribution likely reflects long term selective pressure from macrolide use in pig production. Similarly, tetracycline resistance determinants differed between isolates *tetW* in NS05 and *tetO* in T14 both encoding ribosomal protection proteins. Tetracycline resistance is one of the most commonly reported resistance traits in *S. suis* worldwide and is strongly associated with pig antimicrobial usage [24,39]. The presence of distinct *tet* genes within isolates from the same host population supports multiple independent horizontal transfer events. Interestingly, vancomycin-associated genes (*vanY* within *vanG* and *vanB* associated clusters) were detected, particularly in T14. Although glycopeptide resistance operons are rarely functional in *S. suis*, remnants or partial clusters have been described and are thought to originate from horizontal acquisition [40]. Functional expression of these clusters requires further phenotypic confirmation. The detection of efflux-associated genes *patA* and *patB* further suggests potential multidrug resistance mechanisms, as efflux systems are known contributors to reduced susceptibility across multiple antimicrobial classes [41]. Comparative analysis of the capsular polysaccharide biosynthesis (*cps*) locus revealed conserved upstream regulatory regions with divergence concentrated in the central variable region. The *cps* locus is a critical virulence determinant in *S. suis*, and variations in glycosyltransferase genes are directly associated with serotype specificity and antigenic diversity [42,43]. Structural variation within the *cps* cluster likely reflects recombination or horizontal gene transfer events, contributing to capsular diversity and potential immune evasion. Such mosaic *cps* architectures have been described previously and are considered drivers of serotype evolution [44]. Functional annotation confirmed the presence of complete capsule biosynthesis pathways, including glycosyltransferases, sugar-modifying enzymes, and ABC transporter components, consistent with intact capsule export systems. Capsule production is essential for resistance to phagocytosis and complement-mediated killing and remains a key virulence factor in invasive *S. suis* infections [33,42].

The average nucleotide identity (ANI) value of 95.06% between NS05 and T14 lies at the accepted species boundary threshold (95–96%) [45], confirming that both isolates belong to *S. suis* while representing genetically distinct lineages. The relatively limited proportion of shared orthologous fragments further supports the presence of an expanded accessory genome in T14. This observation aligns with previous reports describing an open pan-genome structure in *S. suis*, particularly among non-serotype 2 isolates and colonizing strains [37,46]. Collectively, these findings highlight the genomic plasticity of *S. suis* colonizing the porcine upper respiratory tract. The detection of multidrug resistance determinants and cps variability underscores the role of nasal and tonsillar niches as reservoirs of genetic diversity and AMR genes. Given the zoonotic potential of *S. suis* and the increasing concern regarding antimicrobial resistance, continued genomic surveillance of colonizing strains is warranted to better understand the emergence of invasive and resistant lineages.

## 4. Materials and Methods

### 4.1. Sample Collection

The sample size was determined using a formula for estimating true prevalence available through Epitools (https://epitools.ausvet.com.au (accessed on 1 January 2026)). Due to variability in reported prevalence rates of *S. suis*, an expected prevalence of 18.2% was adopted based on Kittiwan et al. (2022) [47]. The calculation incorporated a test sensitivity of 95%, specificity of 90%, a desired precision of 10%, and a confidence level of 90%.

**Figure 10:**
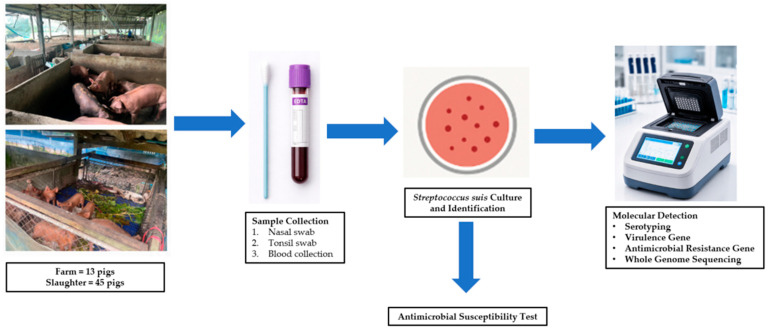
Experimental workflow for antimicrobial susceptibility testing and molecular characterization of *S. suis*.

A total of 58 pigs were sampled between February and May 2026 in Nakhon Si Thammarat Province, southern Thailand. Thirteen pigs from thirteen commercial pig farms and 45 pigs from ten slaughter batches at local abattoirs were included in the study. Farms and abattoirs were selected based on accessibility and owner consent during the study period. A nasal swab and a blood sample were collected from each farm pig, whereas a nasal swab, a tonsil swab, and a blood sample were collected from each abattoir pig. In total, 161 samples were collected, consisting of 58 nasal swabs, 45 tonsil swabs, and 58 blood samples. Swab samples were collected using sterile swabs and placed in Stuart transport medium, while blood samples were collected from the jugular vein into sterile EDTA tubes. All specimens were transported to the laboratory within 4 h of collection for further processing. The overall study workflow is presented in Figure 10.

### 4.2. Bacterial Identification and DNA Extraction

The collected samples were cultured on Todd Hewitt agar (Oxoid Ltd., Hampshire, UK) supplemented with 5% sterile defibrinated sheep blood (Thermo Fisher Scientific, Auckland, New Zealand), 0.005% sodium azide (Sigma-Aldrich, St. Louis, MO, USA), 0.003% nalidixic acid (Sigma-Aldrich, St. Louis, MO, USA), 0.002% colistin (Sigma-Aldrich, St. Louis, MO, USA), and 0.0002% crystal violet (Sigma-Aldrich, St. Louis, MO, USA), and incubated at 37 °C in an atmosphere containing 5% CO_2_ for 24–48 h. Colonies exhibiting typical *S. suis* morphology were selected (small colony with alpha-hemolysis) and subcultured to obtain pure isolates on the same agar. Preliminary identification was carried out based on Gram staining, catalase reaction, and hemolysis pattern and then confirmed as *S. suis* using a polymerase chain reaction. For DNA extraction, approximately ten colonies of *S. suis* grown on Todd Hewitt agar were suspended in 100 µL of deionized water and boiled for 10 min. DNA was extracted by using a commercial kit (Geneaid, New Taipei City, Taiwan). PCR amplification was performed using a set of *S. suis* specific primer targeting glutamate dehydrogenase (*gdh*) gene: JP4 (5′-GCAGCGTATTCTGTCAAACG-3′) and JP5 (5′-CCATGGACAGATAAAGATGG-3′: 695 bp) for the identification of the strains [48]. The PCR conditions consisted of an initial denaturation at 94 °C for 5 min, followed by 35 cycles of denaturation at 94 °C for 1 min, annealing at 55 °C for 1 min, and extension at 72 °C for 1 min. A final extension step was carried out at 72 °C for 7 min. *S. suis* DMST18783 was used as the positive control, while nuclease-free water was included as a negative control in each PCR run.

### 4.3. Antibodies Against S. suis Serotype 2 Determination

Serum samples of pigs were used to determine immunoglobulin G against *S. suis* serotype 2 with indirect enzyme linked immunosorbent assay (iELISA) using a commercial kit (ECALBIO^®^, Wuhan, China). The protocol was followed the manufacturer instruction, and some steps were modified following a previous study [49]. Briefly, a total of 5 µL of each serum samples, positive control, and negative control were individually diluted in 195 µL of sample diluent solution. Then, the 96-well microtiter plate coated with whole cell lysate of *S. suis* serotype 2 was prepared by 1X wash buffer washing before testing. A total of 100 µL of tested samples and controls were added to the prepared well (triplication per sample) and then incubated the plate at 37 °C for 30 min. The incubated samples were removed and then each well was washed 5 times using 1X wash buffer. Anti-porcine immunoglobulin conjugated horseradish peroxidase was added into each well (100 µL per well) and incubated at 37 °C for 30 min. After that, the conjugate solution was removed, and the plate was washed 5 times with 1X wash buffer. A total of 100 µL substrate solution was added into each well and incubated at 25 °C for 15 min, then 50 µL stop solution was added into each well. The plate was immediately read the optical density at 360 nm (OD630) using a spectrophotometer (Multiskan SkyHigh Microplate Spectrophotometer, Thermo Fisher Scientific Inc., Waltham, MA, USA) and the SkanIt Software version 6.1RE (Thermo Scientific SkanIt Software, Thermo Fisher Scientific Inc., Waltham, MA, USA). The validation of positive and negative control was analyzed using this calculation: the average OD630 of positive control—the average OD630 of negative control ≥ 0.3. Then, the average OD of each sample was calculated for S/P value using this formula: the average OD630 of each sample/the average OD630 of positive control. The sample that revealed S/P ≥ 0.2 was considered as a seropositive sample.

### 4.4. Antimicrobial Susceptibility Testing and Multidrug Resistance Profile

The antimicrobial susceptibility profiles of sixty *S*. *suis* isolates were determined using the disc diffusion method. The confirmed isolates were cultured overnight at 37 °C in 5% CO_2_ on blood agar supplemented with 5% sheep blood. Colonies from overnight cultures were suspended in 1× phosphate-buffered saline (PBS), and the turbidity of each suspension was adjusted to the 0.5 McFarland standard, corresponding to approximately 1 × 10^8^ CFU/mL. The standardized bacterial suspensions were inoculated onto Mueller–Hinton agar plates supplemented with 5% sheep blood. Antibiotic discs were aseptically placed onto each plate using sterile forceps.

Ten antimicrobial agents were selected for testing, representing major antimicrobial classes commonly used in swine production and veterinary medicine in Thailand, as well as agents frequently included in previous surveillance studies of *S. suis* and other porcine pathogens. These included β-lactams (ceftriaxone, ampicillin, cefepime), macrolides/lincosamides (erythromycin, azithromycin, clindamycin), tetracyclines (tetracycline), phenicols (chloramphenicol), fluoroquinolones (levofloxacin), and glycopeptides (vancomycin). The following antimicrobial agents were tested: ceftriaxone 30 µg (CRO), ampicillin 10 µg (AMP), vancomycin 30 µg (VA), erythromycin 15 µg (E), cefepime 30 µg (FEP), azithromycin 15 µg (AZM), levofloxacin 5 µg (LEV), clindamycin 2 µg (DA), tetracycline 30 µg (TE), and chloramphenicol 30 µg (C) (Oxoid, Basingstoke, UK). Plates were incubated at 37 °C in 5% CO_2_ for 18 h. The diameters of inhibition zones were measured and interpreted as susceptible (S), intermediate (I), or resistant (R) according to Clinical and Laboratory Standards Institute (CLSI, 2019) interpretive criteria for fastidious organisms. Because specific CLSI breakpoints are not available for all antimicrobial agents tested against *S. suis*, interpretive criteria for closely related fastidious streptococci were applied where appropriate [50].

### 4.5. Detection of Antimicrobial Resistance Gene

A subset of thirty *S. suis* isolates was randomly selected from the 60 PCR confirmed isolates for antimicrobial resistance gene detection by conventional PCR. The selected isolates included representatives from both nasal and tonsillar samples and encompassed a range of antimicrobial susceptibility profiles. Because only a subset of isolates was examined, the prevalence of resistance genes should be interpreted with caution. Following the antimicrobial susceptibility testing, genes encoding tetracycline resistance proteins (*tetO* and *tetM*), erythromycin ribosomal methylases (*ermA* and *ermB*), and the macrolide efflux pump (*mefA*) were detected by PCR. The primer pairs used for amplification of these resistance genes are listed in Table 9. PCR amplification was carried out under the following conditions: an initial denaturation at 95 °C for 2 min, followed by 30 cycles of denaturation at 95 °C for 1 min, annealing and extension at gene-specific temperatures for 1 min (53 °C for *tetO*, 52 °C for *ermA* and *tetM*, 50 °C for *ermB*, and 54 °C for *mefA*), and a final extension at 72 °C for 5 min [51,52]. No reference strain was used as a positive control. The expected amplicon sizes were determined according to previously published primer sets, and nuclease-free water was included as a negative control in each PCR assay.

### 4.6. Molecular Typing

*S. suis* isolates were determined their molecular typing using multiplex PCR with sets of serotype specific primers. The molecular typing determination was targeted to serotypes 1, 1/2, 2, 5, 7, 8, 9, 14, and 16. The primers used in this study targeted the capsule polysaccharide (CPS) biosynthesis genes specific to each serotype, including *cps1J* (for serotypes 1 and 14), *cps2J* (for serotypes 2 and 1/2), *cps5N* (for serotype 5), *cps7H* (for serotype 7), *cps8H* (for serotype 8), *cps9H* (for serotype 9), and *cps16K* (for serotype 16), together with the *gdh* gene, which serves as a universal marker for all serotypes [27,28]. The PCR mixture composition followed the protocol described by Kerdsin et al. [24]. The thermal cycling profile consisted of an initial denaturation at 95 °C for 3 min, followed by 30 cycles of denaturation at 95 °C for 20 s, annealing and extension at 62 °C for 90 s, and a final extension at 72 °C for 5 min (Table 10).

### 4.7. Detection of Virulence Genes in S. suis Isolates

Genomic DNA was isolated from 60 *S. suis* isolates using a bacterial genomic DNA kit (Geneaid, New Taipei City, Taiwan) following the manufacturer’s instructions. The presence of *arcA*, *bay046*, *epf*, *hyl*, *mrp*, and *sly* genes was assessed by conventional and multiplex PCR [48,53,54,55,56,57]. The oligonucleotide primer sequences, expected amplicon sizes, and thermocycling conditions are provided in Table 11. Each 25 µL PCR contained 2.5 mM deoxynucleotide triphosphates (dNTPs), 10X PCR buffer (Fermentas, Vilnius, Lithuania), 2.5 mM MgCl_2_, 100 pmol of each forward and reverse primer (Bio Basic Inc., Toronto, ON, Canada), 1.5 U of *Taq* DNA polymerase (Fermentas), and 25 ng of template DNA in a total volume of 25 µL, adjusted with ultrapure (DNase-RNase-Proteinase free). The PCR conditions consisted of an initial denaturation at 94 °C for 4 min, followed by 35 cycles of denaturation at 94 °C for 30 s, annealing at 54 °C for 1 min, and extension at 72 °C for 1 min, with a final extension at 72 °C for 7 min. No reference strain was used as a positive control. The expected amplicon sizes were determined according to previously published primer sets, and nuclease-free water was included as a negative control in each PCR assay. Amplification products were separated by electrophoresis on a 1.2% agarose gel (Axygen Biosciences, Union City, CA, USA) using Tris-Borate-EDTA (TBE) buffer and visualized under UV light after staining with ethidium bromide (Sigma Chemical Co., St. Louis, MO, USA) (Table 11).

### 4.8. Whole Genome Sequencing of S. suis

Two *S. suis* isolates (NS05 and T14) were selected for whole genome sequencing based on their multidrug resistance (MDR) profiles and the highest multiple antibiotic resistance (MAR) indices among all isolates examined. In addition, the isolates represented different anatomical sites and originated from different pigs, with NS05 obtained from a nasal swab and T14 recovered from a tonsillar swab. These isolates were selected to provide preliminary comparative genomic insights into MDR strains from distinct host niches. Genomic DNA was extracted from the selected isolates using a commercial DNA extraction kit (Geneaid, New Taipei City, Taiwan). DNA concentration was quantified according to Illumina sequencing requirements, and samples were diluted in 10 mM Tris buffer (pH 8.0) to achieve a final concentration of ≥10 nM in 10 µL. DNA quality and purity were assessed by measuring A260/280 and A260/230 ratios using a NanoDrop ND-1000 spectrophotometer (Thermo Fisher Scientific, Wilmington, Delaware, USA). Whole-genome sequencing (WGS) was performed using the Illumina MiSeq platform (Illumina Inc., San Diego, CA, USA).

### 4.9. Bioinformatic Analysis

Raw sequence data were quality-assessed using FastQC [57] and adapter trimming was performed with Trimmomatic [58] to remove low-quality bases and residual adapter sequences. Cleaned reads were assembled de novo using SPAdes v3.15 [59], and the quality of the assemblies was verified using QUAST [60]. Species confirmation was conducted using KmerFinder v3.2 (https://cge.food.dtu.dk/services/KmerFinder/, accessed date 20 April 2026) [61]. Multilocus sequence typing (MLST) was performed via the PubMLST database (https://pubmlst.org/, accessed date 20 April 2026) based on the *S. suis* scheme consisting of seven housekeeping genes (*aroA*, *cpn60*, *dpr*, *gki*, *mutS*, *recA*, and *thrA*) [62]. Antimicrobial resistance genes (ARGs) were identified using ResFinder v4.1 (https://cge.food.dtu.dk/services/ResFinder/, accessed date 20 April 2026) [63] with minimum thresholds of 90% sequence identity and 60% gene length coverage. Mobile genetic elements (MGEs), including integrative and conjugative elements, insertion sequences, and transposons, were characterized using MobileElementFinder v1.0.3 (https://cge.food.dtu.dk/services/MobileElementFinder/, accessed date 20 April 2026) [64]. Virulence-associated genes were identified using the Virulence Factor Database (VFDB) (http://www.mgc.ac.cn/VFs/, accessed date 20 April 2026). Searches were conducted using BLASTp (BLAST+ version 2.15.0) with a significance threshold of E-value ≤ 1 × 10^−10^ and query coverage > 40% [65].

### 4.10. Statistical Analysis

All statistical analyses were performed using R version 4.3.0. The prevalence of *S. suis* and antimicrobial resistance genes was calculated as proportions, and corresponding 95% confidence intervals (CIs) were estimated using the Wilson score method. Differences in prevalence among sample types and among antimicrobial resistance genes were assessed using the Fisher Freeman Halton exact test, which is appropriate for categorical data with small sample sizes. Serological results were expressed as mean ± standard deviation (SD). A *p*-value of less than 0.05 was considered statistically significant.

## 5. Conclusions

This study confirms that *S. suis* is commonly present in the porcine upper respiratory tract, particularly in nasal and tonsillar niches, where it serves as an important reservoir for transmission. The high prevalence of multidrug resistance, including widespread tetracycline and macrolide resistance determinants, reflects substantial antimicrobial selective pressure in pig production and raises both veterinary and public health concerns, especially given the predominance of zoonotic serotypes 2 and 1/2. Whole-genome analysis revealed notable genomic diversity, including accessory genome expansion and variability in the cps locus, highlighting the species’ genomic plasticity and capacity for horizontal gene transfer. Collectively, these findings emphasize the importance of continued genomic surveillance and responsible antimicrobial stewardship to mitigate the emergence and spread of invasive and resistant *S. suis* lineages within a One Health framework.

## Figures and Tables

**Table 1:** Number and percentage of positive isolates by sample type (*n* = 176).

Sample	Number of Isolates	Number of Positive for *S. suis* (%)	95% CI
Tonsil Swab	76	26, 34.2	24.3–45.5%
Nasal Swab	89	34, 38.2	28.8–48.5%
Blood	11	0, 0	0–25.9%
Total	176	60, 34.1	27.4–41.3%

Abbreviation: CI = Confidence Interval, Positive isolates represent culture-positive samples confirmed as *S. suis* by PCR.

**Table 2:** The percentage of susceptible (S), intermediate (I), and resistant (R) isolates from nasal and tonsil swabs.

	Nasal Swab			Tonsil Swab		
	S	I	R	S	I	R
**CRO**	76.47	0	23.53	69.23	0	30.77
**AMP**	73.53	0	26.47	69.23	0	30.77
**VA**	23.53	0	76.47	15.38	0	84.62
**E**	35.29	20.59	14.71	50	7.69	42.31
**FEP**	70.59	0	29.41	65.38	0	34.62
**AZM**	44.12	20.59	35.29	61.54	0	38.46
**LEV**	20.59	2.94	76.47	19.23	3.85	76.92
**DA**	67.65	5.88	26.47	76.92	0	23.08
**TE**	55.88	14.71	29.41	69.23	7.69	23.08
**C**	8.82	2.94	88.24	11.54	0	88.46

**Table 3:** Prevalence of antimicrobial resistance genes detected among isolates (n = 30).

Antimicrobial Resistance Genes	Number of Positive (%)	*p*-Value
*tetO*	24 (80%)	*p* = 0.045
*tetM*	27 (90%)	
*ermA*	17 (56.67%)	
*ermB*	21 (70%)	
*mefA*	23 (76.67%)	

**Table 4:** Serotype distribution of *S. suis* isolates from nasal swab and tonsil swab (*n* = 60).

Serotype	Number (%) of Positives by Specimens		Total (*n* = 60)
	Nasal Swab (*n* = 34)	Tonsil Swab (*n* = 26)	
1, 14	4 (11.76)	6 (23.07)	10 (16.67)
2, 1/2	12 (35.29)	6 (23.07)	18 (30.00)
5	6 (17.64)	2 (7.69)	8 (13.33)
7	0 (0)	0 (0)	0 (0)
8	9 (26.47)	4 (15.38)	13 (21.67)
9	0 (0)	0 (0)	0 (0)
16	1 (2.94)	2 (7.69)	1 (1.67)

**Table 5:** Prevalence of virulence genes in *S. suis* isolates obtained from nasal and tonsillar samples (*n* = 60).

Virulence Genes	Number (%) of Positives by Specimens (*n* = 60)	
	Nasal Swab (*n* = 34)	Tonsil Swab (*n* = 26)
*bay046*	32 (94.12)	24, 92.31%
*arcA*	27, 79.41%	15, 57.69%
*epf*	0, 0%	0, 0%
*mrp*	2, 5.88%	1, 3.85%
*hly*	0, 0%	0, 0%
*sly*	0, 0%	0, 0%

**Table 6:** The percentage of virulence associated genes detected among *S. suis* serotypes (*n* = 60).

	Serotypes 1 and 14	Serotypes 2 and 1/2	Serotype 5	Serotype 7	Serotype 8	Serotype 9	Serotype 16
*bay046*	15	30	13.33	0	21.67	0	5
*arcA*	10	21.67	6.67	0	11.67	0	1.67
*epf*	0	0	0	0	0	0	0
*mrp*	0	1.67	0	0	0	0	0
*hly*	0	0	0	0	0	0	0
*sly*	0	0	0	0	0	0	0

**Table 7:** Genome Assembly Detail of two *S. suis* isolates by WGS.

Strain	NS05	T14
Contigs	92	78
GC Content	41.03%	39.67%
Plasmids	Not found	Not found
Contig L50	12	7
Genome Length	2,183,810 bp	3,846,008 bp
Contig N50	68,551	168,725

**Table 8:** Antimicrobial Resistance Genes by WGS.

AMR Mechanism	NS05	T14
Antibiotic inactivation enzyme	*APH(3′)-III/APH(3′)-IV/APH(3′)-VI/APH(3′)-VII*, *CatA8 family*	*ANT(6)-I*, *ANT(9)-I*
Antibiotic target in susceptible species	*Alr*, *Ddl*, *EF-G*, *EF-Tu*, *folA*, *Dfr*, *folP*, *gyrA*, *gyrB*, *IsotRNA*, *kasA*, *MurA*, *rpoB*, *rpoC*, *S10p*, *S12p*	*Alr*, *Ddl*, *EF-G*, *EF-Tu*, *folA*, *Dfr*, *folP*, *gyrA*, *gyrB*, *Iso-tRNA*, *kasA*, *MurA*, *rpoB*, *rpoC*, *S10p*, *S12p*
Antibiotic target modifying enzyme	*Erm(B)*, *RlmA(II)*	*Erm(B)*, *RlmA(II)*
Antibiotic target protection protein	*Tet(W)*	*Tet(O)*
Antibiotic target replacement protein	*FabK*	*FabK*
Gene conferring resistance via absence	*gidB*	*gidB*
Protein altering cell wall charge conferring antibiotic resistance	*PgsA*	*GdpD*, *MprF*, *PgsA*
Regulator modulating expression of antibiotic resistance genes	*LiaF*, *LiaR*, *LiaS*	*LiaF*, *LiaR*, *LiaS*

**Table 9:** Primer sequences and amplicon sizes for detection of antibiotic resistance genes.

Primer	Sequence	Size (bp)	References
*tetM*	GTGGAGTACTACATTTACGAG (forward)	406	51
	GAAGCGGATCACTATCTGAG (reverse)		
*tetO*	GCGGAACATTGCATTTGAGGG (forward)	515	
	CTCTATGGACAACCCGACAGAAG (reverse)		
*ermA*	TCAGGAAAAGGACATTTTACC (forward)	190	
	ATACTTTTTGTAGTCCTTCTT (reverse)		
*ermB*	GGTAAAGGGCATTTAACGAC (forward)	639	52
	CGATATTCTCGATTGACCCA (reverse)		
*mefA*	AGTATCATTAATCACTAGTGC (forward)	348	
	TTCTTCTGGTACTAAAAGTGG (reverse)		

**Table 10:** Primers used for PCR amplification of capsular polysaccharide (cps) genes and the *gdh* gene, including nucleotide sequences, expected amplicon sizes, and references.

Primer	Sequence	Size (bp)	References
*cps1J*	GGCGGTCTAGCAGATGCTCG (forward)	675	53
	GCGAACTGTTAGCCATGAC (reverse)		
*cps2J*	GTTGAGTCCTTATACACCTGTT (forward)	459	
	CAGAAAATTCATATTGTCCACC (reverse)		
*cps5N*	TGATGGCGGAGTTTGGGTCGC (forward)	166	
	CGTAACAACCGCCCCAGCCG (reverse)		
*cps7H*	AGCTCTAACACGAAATAAGGC (forward)	251	
	GTCAAACACCCTGGATAGCCG (reverse)		
*cps8H*	ATGGGCGTTGGCGGGAGTTT (forward)	320	
	TTACGGCCCCCATCACGCTG (reverse)		
*cps9H*	GGCTACATATAATGGAAGCCC (forward)	390	
	CCGAAGTATCTGGGCTACTG (reverse)		
*cps16K*	TGGAGGAGCATCTACAGCTCGGAAT (forward)	202	
	TTTGTTTGCTGGAATCTCAGGCACC (reverse)		
*gdh*	TTCTGCAGCGTATTCTGTCAAACG (forward)	695	54
	TGTTCCATGGACAGATAAAGATGG (reverse)		

**Table 11:** PCR primers and amplification conditions for detection of selected virulence-associated genes.

Primer	Sequence	Size (bp)	References
*gdh*	GCAGCGTATTCTGTCAAACG (forward)	688	48
	CCATGGACAGATAAAGATGG (reverse)		
*cps2*	TGATAGTGATTTGTCGGGAGGG (forward)	557	55
	GAGTATCTAAAGAATGCCTATTG (reverse)		
*epf*	ATCTACTGGGTATCCTTCTGC (forward)	626	56
	CTATCTGGATCTGTGATTGGA (reverse)		
*mrp*	TGCTGAAAATACGAGTGC (forward)	970	
	TGCCADCATAATCATACCC (reverse)		
*sly*	ACTCTATCACCTCATCCGC (forward)	1400	
	ATGAGAAAAAGTTCGCACTTG (reverse)		
*hyl*	CTCAGATGAAAGCCTTTCTA (forward)	1290	
	TTTGTCCTTGGTCGTTGTC (reverse)		
*arcA*	GATGCCTTTGCTCAAGCTCT (forward)	441	
	TTTCACGGTTCCGTGTTTCT (reverse)		
*bay046*	ATGCCACGGATTACCTTCCC (forward)	253	
	CCGTCTCCTTAATGATCCGC (reverse)		

## Data Availability

The data presented in this study are available in the article.

## Supplementary Materials

The following supporting information can be downloaded at: https://www.mdpi.com/article/10.3390/antibiotics15070660/s1, Table S1. Antimicrobial resistance patterns of *S. suis* isolates based on disc diffusion results.
