# Supplementary material for: Antimicrobial Resistance, Serotypes, Virulence Gene Profiles, and Molecular Characterization of Streptococcus suis Isolated from Healthy Pigs in Thailand
Source: Antibiotics (Basel). 2026 Jul 3;15(7):660. doi: 10.3390/antibiotics15070660 (PMC13404316; doi:10.3390/antibiotics15070660)
Supplement: Supplementary file 1 [file antibiotics-15-00660-s001.zip › antibiotics-4385011-supplementary.pdf]

Table S1. Antimicrobial resistance patterns of *S. suis* isolates based on disc diffusion results.

No.	MDR Pattern	Isolates (n)	Drug classes
1	CRO-AMP-VA-FEP-DA-TE	1	5
2	CRO-AMP-VA-E-FEP-AZM-LEV-DA-TE	1	7
3	CRO-AMP-VA-E-FEP-LEV-DA-TE	1	7
4	CRO-AMP-VA-E-FEP	2	4
5	CRO-AMP-E-FEP-AZM-DA-TE	4	5
6	E-AZM-LEV-DA-TE	2	4
7	AMP-LEV-DA-TE	1	4
8	E-FEP-AZM-DA-TE	1	4
9	E-AZM-DA-TE	2	3
10	CRO-AMP-VA-E-FEP-AZM-DA-TE	2	6
11	E-AZM-LEV-DA-TE-C	1	5
12	CRO-AMP-E-FEP-AZM-LEV-DA-TE	1	6
13	AMP-E-AZM-DA-TE-C	1	5
14	CRO-AMP-VA-E-FEP-DA-TE	1	6
15	CRO-AMP-E-FEP-AZM-DA-TE	1	5
16	AMP-E-AZM-LEV-DA-TE	1	5
17	AZM-DA-TE	1	3
18	CRO-AMP-FEP-LEV-DA-TE	2	5
19	CRO-AMP-FEP-DA-TE	2	4
20	CRO-AMP-FEP-AZM-DA-TE	1	5
21	CRO-AMP-FEP-DA	1	3
22	CRO-AZM-DA-TE-C	1	5
23	CRO-VA-AZM-DA-TE-C	1	6

No.	MDR Pattern	Isolates (n)	Drug classes
24	AZM-DA-TE-C	2	4
25	CRO-AMP-VA-FEP-AZM-DA	2	5
26	CRO-AMP-VA-FEP-AZM-DA-TE	1	6
27	CRO-E-AZM-DA	2	3
28	CRO-AMP-FEP-AZM-DA	1	4
	Total	39	
